# *Pf*ARID Regulates P. falciparum Malaria Parasite Male Gametogenesis and Female Fertility and Is Critical for Parasite Transmission to the Mosquito Vector

**DOI:** 10.1128/mbio.00578-22

**Published:** 2022-05-31

**Authors:** Sudhir Kumar, Vinay K. Baranwal, Meseret T. Haile, Kenza M. Z. Oualim, Biley A. Abatiyow, Spencer Y. Kennedy, Ashley M. Vaughan, Stefan H. I. Kappe

**Affiliations:** a Center for Global Infectious Disease Research, Seattle Children’s Research Institute, Seattle, Washington, USA; b Molecular Botany Lab, Swami Devanand Post Graduate College, Math-Lar, Deoria, Uttar Pradesh, India; c Department of Pediatrics, University of Washington, Seattle, Washington, USA; d Department of Global Health, University of Washington, Seattle, Washington, USA; Albert Einstein College of Medicine

**Keywords:** ARID, chromatin, differentiation, gametocyte, transmission

## Abstract

Sexual reproduction of Plasmodium falciparum parasites is critical to the spread of malaria in the human population. The factors that regulate gene expression underlying formation of fertilization-competent gametes, however, remain unknown. Here, we report that P. falciparum expresses a protein with an AT-rich interaction domain (ARID) which, in other organisms, is part of chromatin remodeling complexes. P. falciparum ARID (*Pf*ARID) localized to the parasite nucleus and is critical for the formation of male gametes and fertility of female gametes. *Pf*ARID gene deletion (*Pfarid*^–^) gametocytes showed downregulation of gene expression important for gametogenesis, antigenic variation, and cell signaling and for parasite development in the mosquito. Our study identifies *Pf*ARID as a critical nuclear protein involved in regulating the gene expression landscape of mature gametocytes. This establishes fertility and also prepares the parasite for postfertilization events that are essential for infection of the mosquito vector.

## INTRODUCTION

Malaria remains a major cause of mortality and morbidity in developing countries across the world. The disease is caused by *Plasmodium* parasites, with most deaths attributed to infection with Plasmodium falciparum. Malaria parasites are alveolates that reproduce asexually within two hosts: a vertebrate such as humans and a mosquito vector. The *Plasmodium* life cycle also has an obligate sexual phase, which initiates in the vertebrate host and is completed in mosquitoes (1). P. falciparum male and female gametocytes are formed by a subset of asexually replicating parasites and develop over 12 to 14 days as morphologically distinct stages I, II, III, IV, and V within infected red blood cells (RBCs). When male and female stage V gametocytes are taken up by the mosquito during a blood meal, they are activated to form male microgametes and female macrogametes. This process is completed within 10 to 15 minutes, and mature gametes then egress from the infected RBCs. The factors controlling gametocyte activation include a decrease in temperature (2), increase in pH (3), and/or exposure to xanthurenic acid (XA), a metabolite of tryptophan (4). Gametogenesis is also linked to mobilization of intracellular calcium (Ca^2+^) stores, which can regulate Ca^2+^-dependent protein function via protein kinase G (PKG), important for calcium mobilization-1 (ICM1) (4, 5) and Ca^2+^-dependent protein kinase 4 (CDPK4) (6). In *P. falciparum*, Gametogenesis is further regulated by the activity of a perforin-like protein, PPLP2 (7), which in turn is regulated by a patatin-like phospholipase (PATPL1) (8). Microgametes fertilize macrogametes to form a zygote, which transforms into a motile ookinete within 24 h. Ookinetes penetrate the mosquito midgut epithelium and each form an oocyst, which produces sporozoites for transmission to the next host.

In all organisms, cellular differentiation is accompanied by activation and/or repression of specific genes via genetic and epigenetic mechanisms (9–12). Sexual differentiation, including germ cell formation and gametophyte formation is controlled by diverse master regulatory factors across the animal and plant kingdoms, respectively (13–15). Over the past decade, the molecular basis of sexual stage differentiation (gametocytogenesis) in *Plasmodium* has begun to be better understood. It involves hierarchical transcriptional control, where a subset of genes is specifically expressed or predominantly expressed in sexual forms of the parasite (16, 17). Members of the plant-like ApiAP2 family of transcription factors, AP2-G, AP2-G2, and AP2-G5, have been shown to play critical roles in regulating sexual stage commitment and gametocytogenesis in human malaria parasite species (18–20). Mature gametocytes also exhibit large-scale translational repression, which represents a major mechanism that prepares the parasite progeny for postfertilization development (21).

For transcription factors to access DNA information, histone proteins must first be repositioned or evicted from the chromosomes, a function that is performed by ATP-dependent chromatin remodeling complexes such as BAF (BRG1/BRM-associated factor) (mammalian SWI/SNF) complexes, which are composed of 14 to 16 individual protein subunits in human cells (22). These complexes control multiple cellular processes such as cell proliferation, transcriptional activation, differentiation, and chromatin remodeling (23–25). However, chromatin remodeling complexes regulating gametogenesis have not been identified in P. falciparum although, *Plasmodium* gametocytogenesis and gametogenesis likely requires chromatin remodeling to regulate expression of specific genes. In searching for putative epigenetic regulators of gametogenesis, we have identified one AT-rich interaction domain (ARID) domain-containing protein in *Plasmodium*, including the human malaria parasite P. falciparum. ARID is an ancient ~100-amino acid DNA-binding module present in various eukaryotic transcriptional regulators, which can be part of chromatin remodeling complexes (26). Although, ARID proteins were initially named due to their preference for AT-rich target DNA, it is now known that most of the ARID family proteins do not prefer an AT-rich target sequence (27).

Here, we explored the role of P. falciparum ARID (*Pf*ARID) in the parasite life cycle. We show that *Pf*ARID is a nuclear protein and is expressed in asexual and sexual erythrocytic stages. *PfARID* gene deletion parasites (*Pfarid*^–^) showed normal asexual blood-stage replication and differentiated into mature stage V gametocytes. In contrast, *Pfarid*^–^ parasites exhibited a complete block in transmission to mosquitoes. Further analysis revealed that *Pfarid*^–^ parasites suffered a severe defect in microgametogenesis, while macrogametes appeared to form normally. Strikingly, however, genetic crosses with male-only sterile parasite lines and female-only sterile lines demonstrated that ARID is also critical for female gamete fertility. We further show that *Pf*ARID regulates gene expression that is important for gametogenesis but also appears to be a regulator for expression of genes that prepare the parasite for the postfertilization steps that are necessary for mosquito infection.

## RESULTS

### A *Plasmodium* ARID domain-containing protein is expressed in asexual and sexual erythrocytic stages.

**FIG 1:**
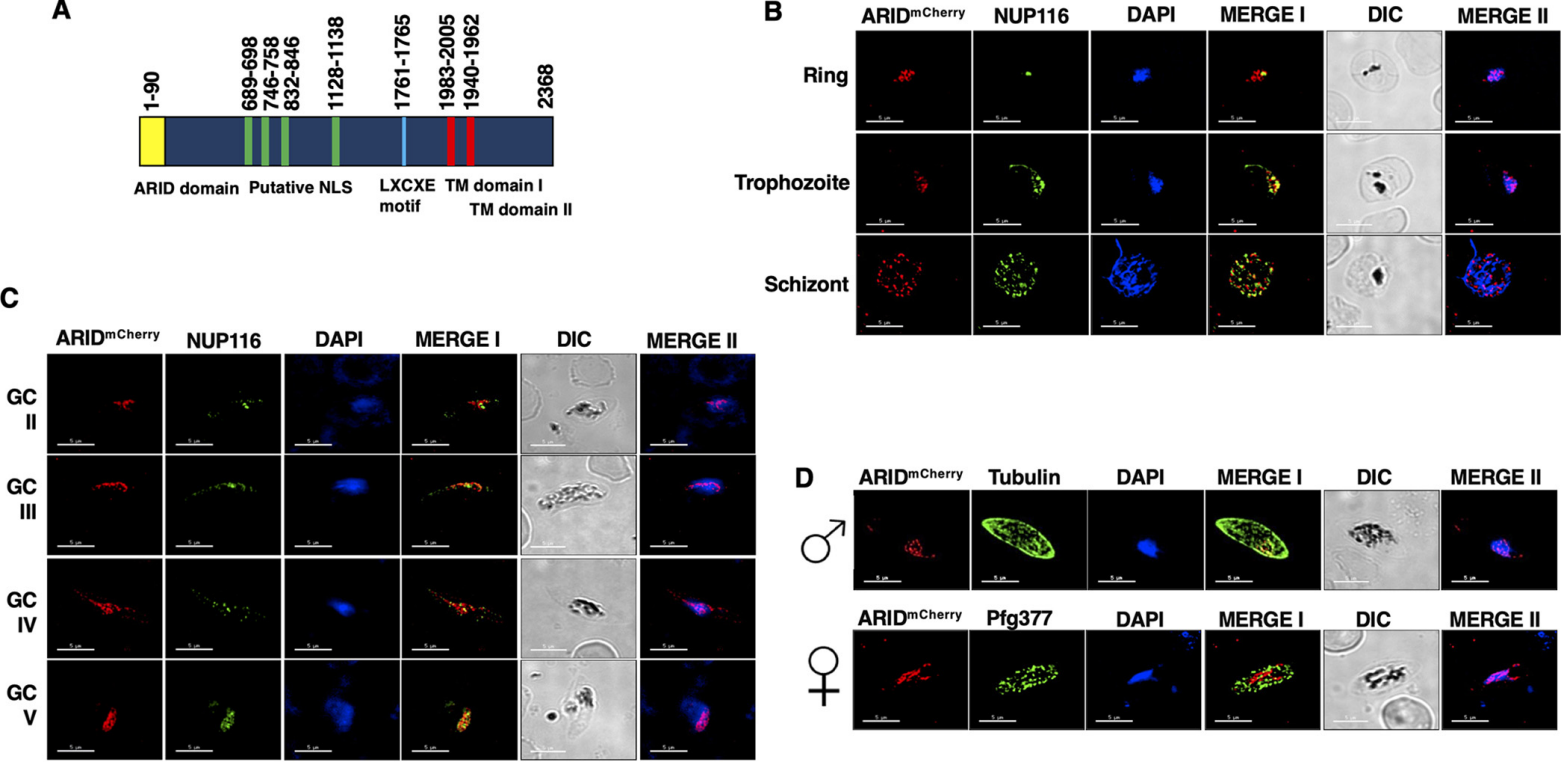
Expression and localization of *Pf*ARID in intraerythrocytic parasite stages. (A) Domain architecture of the P. falciparum ARID protein showing the ARID domain (1 to 90 amino acids [aa]) in yellow, the putative NLS in green, an LXCXE motif in blue, and two transmembrane domains (TM) in red. (B and C) IFAs were performed on asexual (ring, trophozoite, and schizonts) and sexual stages (stage II to V gametocytes) using anti-mCherry antibody (in red) in combination with anti-*Pf*NUP116 antibody (in green). (D) IFAs were performed on sexual stages using an α-mCherry antibody in combination with α-tubulin II (male gametocytes) or α-*Pf*g377 antibodies (female gametocytes). The parasite DNA was stained with DAPI (blue). Scale bar = 5 μm. Images are shown from representative experiments. Merge I, merged image for red and green channels; merge II, merged image for red and DAPI (blue) channels; GC, gametocytes.

To identify ARID orthologs in the Plasmodium falciparum genome, we searched for PFAM domain PF01388 using PlasmoDB v.50, revealing a single gene with an encoded ARID domain, which we have termed *PfARID* (PF3D7_0603600). Predicted amino acid sequence analysis revealed that the ARID domain is located at the N terminus of the protein (Fig. 1A). *Pf*ARID has multiple predicted internal nuclear localization signals (NLS) downstream of the ARID domain and has two predicted transmembrane (TM) domains toward the C-terminal part of the protein (Fig. 1A) The presence of TMs appears unique compared to other ARID proteins, which have no TM domains (28). In addition, *Pf*ARID also shows an LXCXE motif (Fig. 1A) which, in other proteins, has a role in facilitating interaction between the retinoblastoma (RB) tumor suppressor and many cellular proteins (29). To analyze the conservation of ARID among different *Plasmodium* species, we performed an amino acid sequence alignment for P. falciparum, P. vivax (*Pv*), P. berghei (*Pb*), and P. yoelii (*Py*) ARID, which revealed high sequence similarities in their respective ARID domains (Fig. S1A). 3-D structure models generated using SWISS Model (https://swissmodel.expasy.org/) revealed that the P. falciparum ARID domain has closest structural similarity with the human ARID3a protein (Fig. S1B), which is involved in the differentiation of hematopoietic progenitors (30).

10.1128/mbio.00578-22.4 FIG S1 *Pf*ARID conservation and generation of *Pf*ARID^mCherry^ parasites. (A) ARID domain of ARID proteins from P. falciparum (*Pf*ARID), Plasmodium vivax (*Pv*), Plasmodium berghei (*Pb*), and Plasmodium yoelii (*Py*). Conserved residues are in white font on a red background. (B) Homology-based predicted three-dimensional structure of *Pf*ARID. PDB template used 4LJX (*Hs*ARID3A). (C) The schematic for endogenous tagging of the *Pf*ARID locus with mCherry. The pFCL3_ARID_mCherry plasmid has homology regions from 5′ (5′HR) and 3′ (3′HR) of the PfARID locus, single guide RNA-seq (sgRNA), and the human dihydrofolate reductase (hDHFR) locus cloned. (D) Confirmation of *Pf*ARIDm^Cherry^ parasite generation by diagnostic PCR. The oligonucleotides were designed from outside 5′HR and 3′HR and the *PfARID* locus, and their positions are indicated by arrows in panel C. (E) The expected sizes for different sets of PCRs. (F and G) IFAs were performed on sexual stages using α-mCherry antibody in combination with H3K9ac. Parasite nucleus was stained with DAPI (blue). Scale bar = 5 μm. Images are shown from representative experiments. Merge I, merged image for red and green channel; merge II, merged image for red and DAPI (blue) channel; GC, gametocytes. Download FIG S1, TIF file, 2.5 MB. Copyright © 2022 Kumar et al. 2022 Kumar et al. https://creativecommons.org/licenses/by/4.0/ This content is distributed under the terms of the Creative Commons Attribution 4.0 International license.

To analyze expression of *Pf*ARID, a transgenic line with an mCherry tag at the C terminus (PfARID^mCherry^) was generated by double crossover recombination (Fig. S1C to E). Indirect immunofluorescence assays (IFAs) were performed on fixed and permeabilized thin blood-stage culture smears using mCherry antibodies and counterlabeling with an anti-NUP116 antibody, which marks nuclear pores (31). Expression of *Pf*ARID was detected in ring, trophozoite, and schizont stages (Fig. 1B). In each stage ARID partially colocalized with DNA and also appeared to be in close proximity to NUP116. *Pf*ARID expression was also detected in gametocytes, from stage II through stage V (Fig. 1C). In these stages, ARID also partially colocalized with DNA and also appeared to be in close proximity to NUP116. Counterstaining with male (anti-tubulin) or female (anti-Pfg377) gametocyte-specific antibodies revealed that *Pf*ARID is expressed in both male and female gametocytes (Fig. 1D). Further analysis performed using an acetylated histone-specific antibody (H3K9Ac) revealed that *Pf*ARID is mainly expressed in euchromatic regions in stage II to V gametocytes (Fig. S1F and G).

### *Pf*ARID is essential for male gametogenesis.

**FIG 2:**
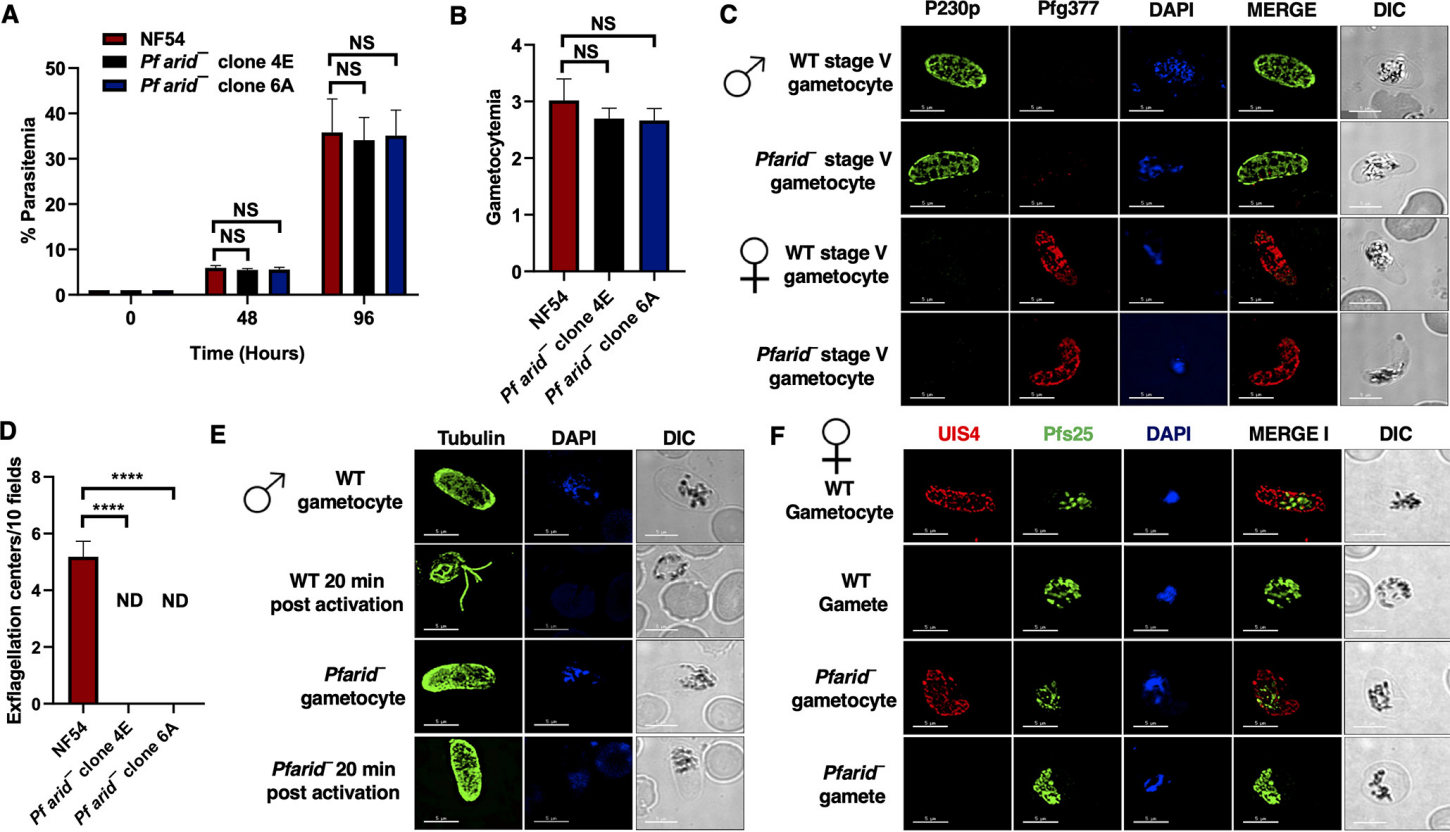
*Pfarid*^–^ parasites grow normally as asexual parasites and undergo gametocytogenesis but fail to form microgametes. (A) Parasite growth rate for WT *Pf*NF54 and *Pfarid*^–^ (clone 4E and 6A) was measured over two erythrocytic cycles using Giemsa-stained smears. Data were averaged from three biological replicates and are presented as the mean ± standard deviation (SD). (B) Gametocytemias for WT *Pf*NF54 and *Pfarid*^–^ (clones 4E and 6A) parasites were measured on day 15 using Giemsa-stained smears. Data were averaged from three biological replicates and are presented as the mean ± SD. (C) IFAs performed for day 15 WT or *Pfarid*^–^ stage V gametocytes (clone 4E) using α-P230p (green), a stage V male-specific marker, and *Pf*g377 (red), a marker for female gametocytes. (D) The number of exflagellation centers (vigorous flagellar beating of microgametes in clusters of RBCs) per field at 15 min postactivation. Data were averaged from three biological replicates and are presented as the mean ± SD. (E) IFAs performed on WT or *Pfarid*^–^ (clone 4E) gametocytes activated for 20 min *in vitro* using α-tubulin II (green), a male-specific marker. α-Tubulin II staining showed male gametes emerging from an exflagellating male gametocyte in WT *Pf*NF54. The *Pfarid*^–^ gametocytes were defective for male gametocyte exflagellation. ND, not detected. (F) IFAs performed on WT *Pf*NF54 and *Pfarid*^–^ (clone 4E) gametocytes activated for 20 min *in vitro* using *Pf*s25 (green), a marker for female gametes, and PfUIS4, marker for the parasitophorous vacuole membrane. Female gametes did not show any defect in egress from the infected RBC. NS, not significant; ND, not detected.

To assess the importance of ARID in the P. falciparum life cycle, the endogenous *PfARID* gene was disrupted using CRISPR/Cas9 methodology (Fig. S2). Gene deletion parasites were confirmed by a set of diagnostic PCRs with oligonucleotides specific for the *PfARID* locus and genomic regions 5′ (upstream) and 3′ (downstream) of the open reading frame (Fig. S2A to C). To analyze a potential function of *Pf*ARID in asexual blood stages, a comparative growth rate assay was set up using two clones of *Pfarid*^–^ (clones 4E and 6A) along with wild-type (WT) *Pf*NF54 parasites, starting with synchronized ring stages. Growth was monitored over two replication cycles using Giemsa-stained thin blood culture smears, which revealed that the growth rate of *Pfarid*^–^ was similar to that of WT *Pf*NF54 parasites (Fig. 2A). This indicated that *Pf*ARID does not have a critical role in asexual blood-stage replication.

10.1128/mbio.00578-22.5 FIG S2 Disruption of the *PfARID* locus via CRISPR/Cas9. (A) The schematic for disrupting the *PfARID* gene. The pFC_ARID_KO plasmid has homology regions from 5′ (5′HR) and 3′ (3′HR) of the *PfARID* locus, single guide RNAseq (sgRNA), and the human dihydrofolate reductase (hDHFR) locus cloned. (B) Confirmation of *PfARID* deletion by diagnostic PCR. The oligonucleotides were designed from outside 5′HR and 3′HR and the *PfARID* locus, and positions are indicated by arrows in panel A. (C) The expected sizes for different sets of PCRs are indicated. Download FIG S2, TIF file, 0.5 MB. Copyright © 2022 Kumar et al. 2022 Kumar et al. https://creativecommons.org/licenses/by/4.0/ This content is distributed under the terms of the Creative Commons Attribution 4.0 International license.

To gain insight into the role of *Pf*ARID in sexual-stage development, we next analyzed the ability of *Pfarid*^–^ to undergo gametocytogenesis. WT *Pf*NF54 and *Pfarid*^–^ (clones 4E and 6A) gametocytes were induced as described previously (32). Gametocytemia was scored for all the cultures on day 15 using Giemsa-stained thin blood culture smears. This revealed that *Pfarid*^–^ parasites were able to develop into mature stage V gametocytes and showed a similar gametocytemia as WT *Pf*NF54 parasites (Fig. 2B). Next, IFAs were performed to analyze the formation of mature male and female stage V gametocytes using anti-P230p and anti-Pfg377 antibodies, respectively. This revealed the apparently normal formation of both male and female *Pfarid*^–^ stage V gametocytes (Fig. 2C).

We next analyzed *Pfarid*^–^ gametogenesis. Day 15 gametocyte cultures for WT *Pf*NF54 and *Pfarid*^–^ (clones 4E and 6A) were activated by addition of O^+^ human serum and a decrease in the temperature from 37°C to room temperature (RT). Activated gametocyte cultures were used to prepare a wet mount, and the emergence of microgametes was assessed using the formation of exflagellation centers in 10 random fields of view under bright-field microscopic illumination at ×400 magnification. Strikingly, and in contrast to WT *Pf*NF54 parasites, we did not observe any exflagellation centers for *Pfarid*^–^ (Fig. 2D), indicating a severe defect in male gametogenesis. To investigate this defect further, IFAs were performed on thin culture smears for WT *Pf*NF54 and *Pfarid*^–^ activated gametocytes 20 min postactivation, and parasites were stained with anti-tubulin antibody, which labels the axoneme of microgametes. The complete absence of microgametes emerging from the male gametocyte body confirmed a severe defect of microgamete formation in *Pfarid*^–^ (Fig. 2E). To analyze formation and egress of female gametes, IFAs were performed using anti-*Pf*s25 antibody (33) and anti-PfUIS4 antibody, which marks the parasitophorous vacuolar membrane (34). These IFAs revealed that *Pfarid*^–^ female gametes formed and egressed from RBCs normally and did not show any apparent morphological defect (Fig. 2F). Taken together, these results show that *Pf*ARID is critical for male gametogenesis.

### *Pf*ARID is also essential for female gamete fertility and for transmission to the mosquito.

**FIG 3:**
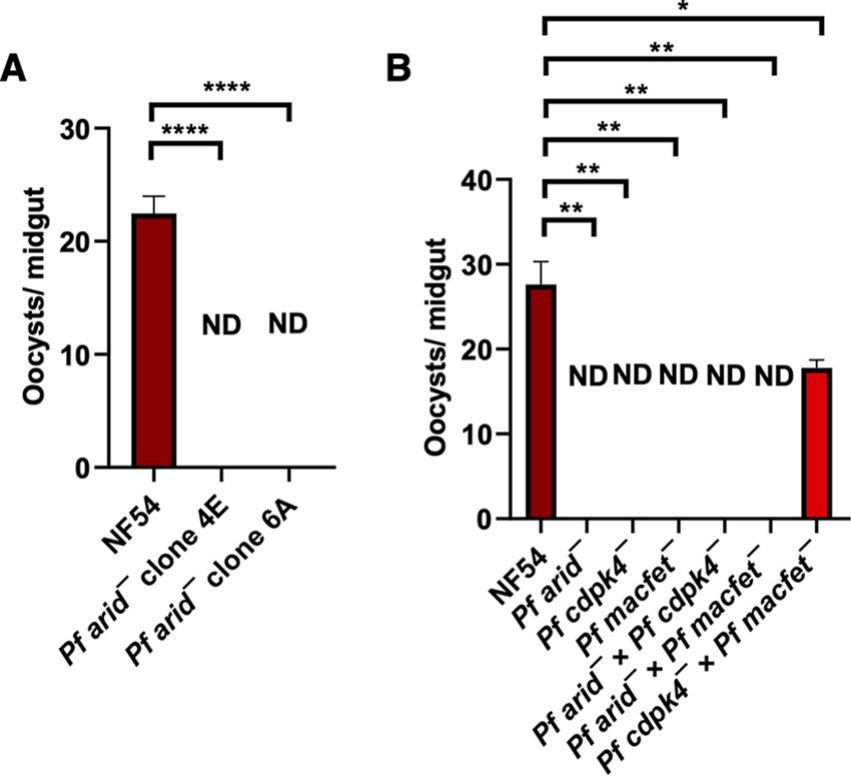
Genetic crosses reveal that *Pf*ARID also regulates fertility of female gametes. (A) WT *Pf*NF54 and *Pfarid*^–^ gametocytes were fed to A. stephensi mosquitoes, and the number of oocysts per mosquito midgut were enumerated on day 7 postfeed. Data were averaged from three biological replicates with a minimum of 50 mosquito guts and are presented as the mean ± standard deviation (SD). *Pfarid*^–^ did not infect the mosquito vector. ND, not detected. (B) Oocyst formation of WT *Pf*NF54, *Pfarid*^–^, *Pfcdpk4*^–^, and *Pfmacfet*^–^ single parasite line feeds and genetic crosses of *Pfarid*^–^ × *Pfcdpk4*^–^, *Pfarid*^–^ × *Pfmacfet*^–^, and *Pfcdpk4*^–^ × *Pfmacfet*^–^. Genetic crosses revealed that the *Pfarid*^–^ did not show productive cross-fertilization with the *Pfmacfet*^–^ parasites (which produce functional males only), and also not with *Pfcdpk4*^–^ parasites (which produce functional females only). This demonstrates that *Pfarid*^–^ parasites suffer a functional defect in both genders. Error bar indicates mean ± SD; *n* = 2. ND, not detected.

After establishing the critical role of *Pf*ARID in male gametogenesis, we next investigated the transmissibility of *Pfarid*^–^ gametocytes to mosquitoes. Infectious blood meals were prepared for WT *Pf*NF54 and *Pfarid*^–^ stage V gametocytes, and gametocytes were fed to mosquitoes using standard membrane feeders. Oocyst stages were analyzed in the mosquito midguts on day 7 postinfection, which revealed a complete absence of oocysts for both *Pfarid*^–^ clones. This was in contrast to WT *Pf*NF54 parasites, which yielded an average oocyst number of ~24/mosquito (Fig. 3A). These results indicated that *Pf*ARID is essential for transmission to the mosquito vector, presumably due to its critical role in male gametogenesis.

To further substantiate the finding that the lack of ARID causes a male-specific defect, we assessed the fertility of male and female *Pfarid*^–^ gametes using genetic crosses with gene deletion parasite lines which either formed only fertile female gametes (*Pfcdpk4*^–^) (6) or only fertile male gametes (*Pfmacfet*^–^) (35). WT *Pf*NF54, *Pfarid*^–^, *Pfcdpk4*^–^, and *Pfmacfet*^–^ gametocytes were generated *in vitro* followed by pairwise mixing and fed to female Anopheles stephensi mosquitoes on day 15 of culture. Genetic crosses were set up as follows: *Pfarid*^–^ × *Pfcdpk4*^–^, *Pfarid*^–^ × *Pfmacfet*^–^, *Pfcdpk4*^–^ × *Pfmacfet*^–^. Mosquitoes were dissected 7 days postfeed to enumerate oocysts in the midgut. As expected, WT *Pf*NF54 gametocytes showed robust mosquito midgut infection, but *Pfarid*^–^, *Pfcdpk4*^–^, and *Pfmacfet*^–^ single line-fed mosquitoes showed no oocysts in the midgut (Fig. 3B). As anticipated, the *Pfarid*^–^ × *Pfcdpk4*^–^ cross showed no mosquito midgut infection, further establishing a complete defect in *Pfarid*^–^ microgametogenesis. Unexpectedly however, the *Pfarid*^–^ × *Pfmacfet*^–^ cross (Fig. 3B) also showed no mosquito midgut oocysts. This indicated that *Pfarid*^–^ parasites suffer an additional severe female fertility defect, which we did not predict based on the apparently normal formation of female gametes (Fig. 2F).

### Widespread transcriptome perturbances in mature *Pfarid^–^* gametocytes.

**FIG 4:**
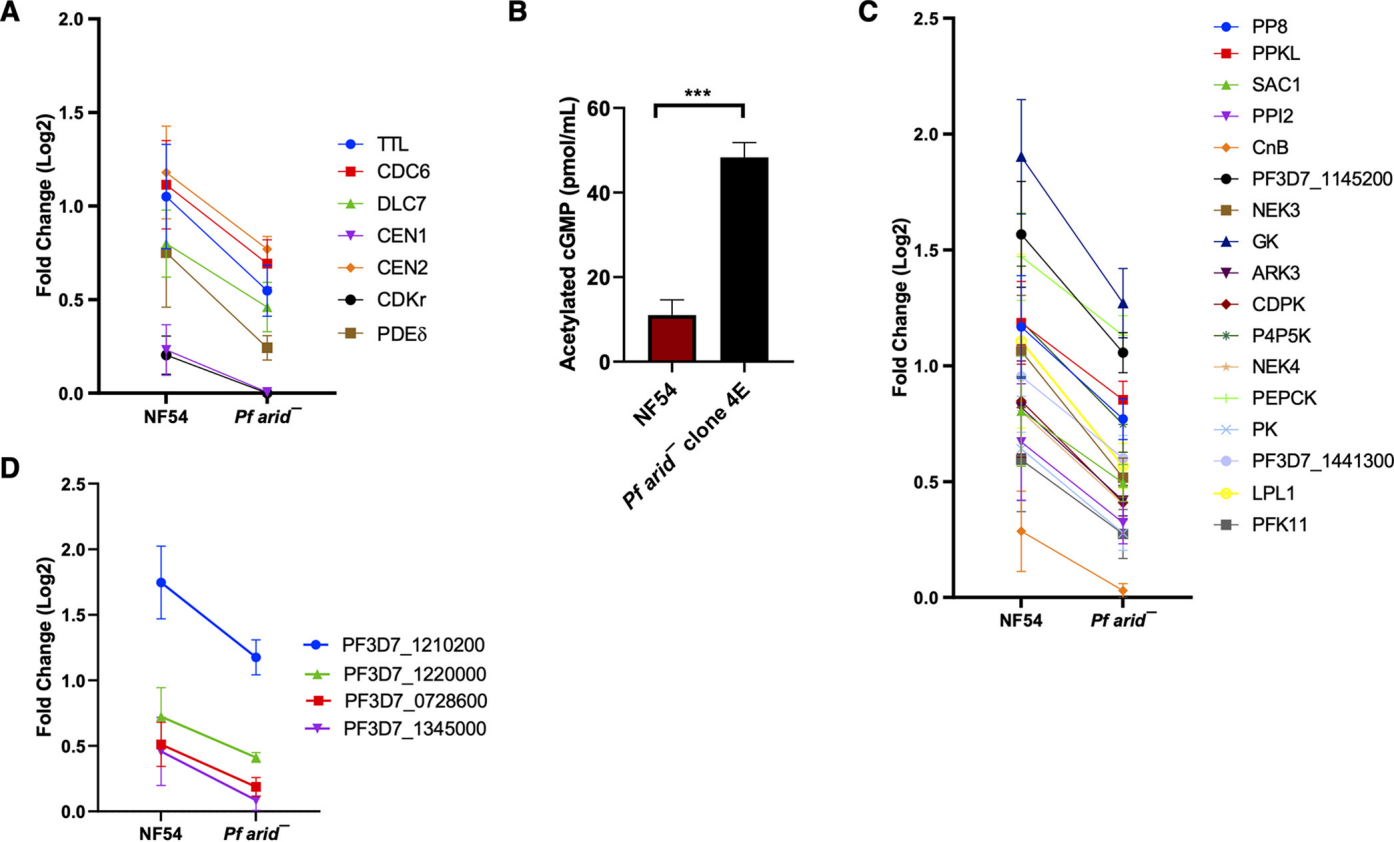
Target-by-target comparison of differential gene expression in WT PfNF54 and *Pfarid*^–^ stage V game oocytes. (A) Graph showing differentially expressed genes (DEGs) from genes predicted to have a role in gametogenesis. Log_2_ fold changes are indicated. (B) Acetylated cGMP levels in day 15 gametocyte extracts prepared for WT *Pf*NF54 and *Pfarid*^–^. Bar graph represents means standard errors of the means. Unpaired *t* test with Welch’s correction was used for statistical analysis. (C) Graph showing DEGs belonging to signaling (kinases, phosphatases, and phospholipases). (D) Graph showing DEGs belonging zinc finger proteins (ZFPs). Log_2_ fold changes are indicated.

Based on the nuclear localization of *Pf*ARID and its association with euchromatic regions, as well as a potential function in chromatin remodeling, we predicted that the lack of *Pf*ARID would cause changes in gene expression. To determine these changes, we performed comparative RNA-seq analysis on WT *Pf*NF54 and *Pfarid*^–^ stage V gametocytes. We focused on this stage, as we anticipated that potential changes in the transcriptome might precede the observed phenotypic defects. RNA-seq was carried out on three biological replicates each for WT *Pf*NF54 and *Pfarid*^–^. This revealed 489 differentially expressed genes (DEGs), of which 78 were upregulated and 411 were downregulated in *Pfarid*^–^ stage V gametocytes (Data set S1A to C). Initially, we curated the DEGs manually to identify genes related to gametogenesis in the malaria parasite and other organisms. We observed that several transcripts encoding proteins such as cGMP-specific 3′,5′-cyclic phosphodiesterase delta (PDE δ), tubulin-tyrosine ligase (TTL), cell division control protein 6 (CDC6), cyclin-dependent kinase regulatory subunit (CDKr), centrin-1 and centrin-2, and flagellar outer arm dynein-associated protein (DLC7) were downregulated in *Pfarid*^–^ (Fig. 4A and Data set S1D). *Pf*PDEδ has been shown to play a role during gametogenesis by regulating cyclic GMP (cGMP) levels (36). We thus measured the levels of cGMP in *Pfarid*^–^ and WT *Pf*NF54 gametocytes, which revealed that cGMP levels were significantly increased in the absence of *Pf*ARID (Figure. 4B). This showed that *Pfarid*^–^ parasites suffer high levels of cGMP during gametocyte development, caused by downregulation of PfPDEδ, and this is deleterious to microgametogenesis.

10.1128/mbio.00578-22.1 DATA SET S1 Summary of differentially expressed genes (DEGs). (S1A) Summary of all DEGs. (S1B) Summary of all upregulated DEGs. (S1C) Summary of all downregulated DEGs. (S1D) Summary of gametogenesis-related DEGs. (S1E) Summary of signaling-related DEGs. (S1F) Summary of DEGs, zinc finger proteins. (S1G) Summary of DEGs, AP2-O targets. (S1H) Summary of DEGs, crystalloid. Download Data Set S1, XLSX file, 0.8 MB. Copyright © 2022 Kumar et al. 2022 Kumar et al. https://creativecommons.org/licenses/by/4.0/ This content is distributed under the terms of the Creative Commons Attribution 4.0 International license.

Interestingly, several signaling molecules, such as kinases and phosphatases and a lysophospholipase (LPL1), were also downregulated in *Pfarid*^–^ (Figure. 4C and Data set S1E). This suggested *Pf*ARID-mediated regulation of expression of genes involved in cellular signaling events, which may be relevant to gametogenesis. The phospholipase PfLPL1 has been shown to regulate neutral lipid synthesis (37). Since a proportion of neutral lipids increase during gametocyte maturation (38), LPL1-mediated generation of neutral lipids may have a role in formation of fertile gametes. Another group of downregulated genes encode zinc finger proteins (ZFPs) (Figure. 4D and Data set S1F). ZFPs are involved in transcriptional regulation, chromatin remodeling, proteostasis, and signal transduction, as well as cell proliferation and differentiation (39, 40). The *Plasmodium* genome encodes 170 putative ZFPs (41). Downregulation of the ZFPs suggested that *Pf*ARID functions upstream of ZFPs and that it might have a regulatory role for expression of these genes during gametogenesis and beyond. This hypothesis is also supported by the recent functional studies in the rodent malaria parasite P. berghei, where female development 4 (FD4) (an ortholog of PF3D7_1220000) is important for completion of female-specific development (42). An additional two ZFPs, PBANKA_1357900 (PF3D7_1345000 ortholog) and PBANKA_0608600 (PF3D7_1210200 ortholog) are critical for the blood to midgut oocyst transition (43), suggesting roles in sexual-stage development.

### *Pfarid*^–^ gametocytes show dysregulation of heterochromatinized gene expression and expression of genes encoding ookinete/crystalloid proteins.

After manual curation, we performed Gene Ontology term enrichment analysis for the DEGs. This analysis revealed that parasite transcripts encoding gene products related to host interactions, host cell, extraorganismal space, and Maurer’s clefts were upregulated in *Pfarid*^–^ stage V gametocytes (Fig. S3A and B). Maurer’s clefts are parasite-derived membranous structures in the infected red blood cell cytosol (44). This group majorly represented multigene family genes such as members of *VAR*, *RIFIN*, and *PHIST* (Data set S1B and 2A and B), which mediate host/parasite interactions. Among the downregulated transcripts, we also found gene terms related to the crystalloid, pellicle, infected host cell surface knobs, inner membrane complex, nucleosome, basal part of the cell, apical part of the cell, signal peptidase complex, and host cell plasma membrane (Fig. S3C D). The majority of the genes belonging to “host cell plasma membrane” included *VAR/RIFIN/PHIST* genes (Fig. S4 and Data set S1C and S2C and D). Since these *VAR/RIFIN/PHIST* multigene families are associated with heterochromatin and heterochromatin protein 1 (HP1) (45–47), their dysregulation may be a result of perturbations of chromatin structure in the *Pfarid*^–^ stage V gametocytes.

10.1128/mbio.00578-22.2 DATA SET S2 Gene Ontology enrichment analysis of DEGs. (S2A) GO term enrichment analysis (molecular function) of upregulated DEGs. (S2B) GO term enrichment analysis (cellular component) of upregulated DEGs. (S2C) GO term enrichment analysis (molecular function) of downregulated DEGs. (S2D) GO term enrichment analysis (cellular component) of downregulated DEGs. Download Data Set S2, XLSX file, 0.02 MB. Copyright © 2022 Kumar et al. 2022 Kumar et al. https://creativecommons.org/licenses/by/4.0/ This content is distributed under the terms of the Creative Commons Attribution 4.0 International license.

10.1128/mbio.00578-22.6 FIG S3 Gene Ontology term enrichment analysis. (A) Molecular function and (B) cellular component terms of upregulated genes. (C) Molecular function and (D) cellular component terms of downregulated genes. Download FIG S3, TIF file, 0.7 MB. Copyright © 2022 Kumar et al. 2022 Kumar et al. https://creativecommons.org/licenses/by/4.0/ This content is distributed under the terms of the Creative Commons Attribution 4.0 International license.

10.1128/mbio.00578-22.7 FIG S4 *Pf*ARID regulates transcription of the heterochromatin-related genes. Heterochromatin-associated gene families with significant dysregulation in *Pfarid*^–^. Geometric mean fold changes and *P* values (two-sided, one sample *t* test) are indicated. Download FIG S4, TIF file, 0.4 MB. Copyright © 2022 Kumar et al. 2022 Kumar et al. https://creativecommons.org/licenses/by/4.0/ This content is distributed under the terms of the Creative Commons Attribution 4.0 International license.

**FIG 5:**
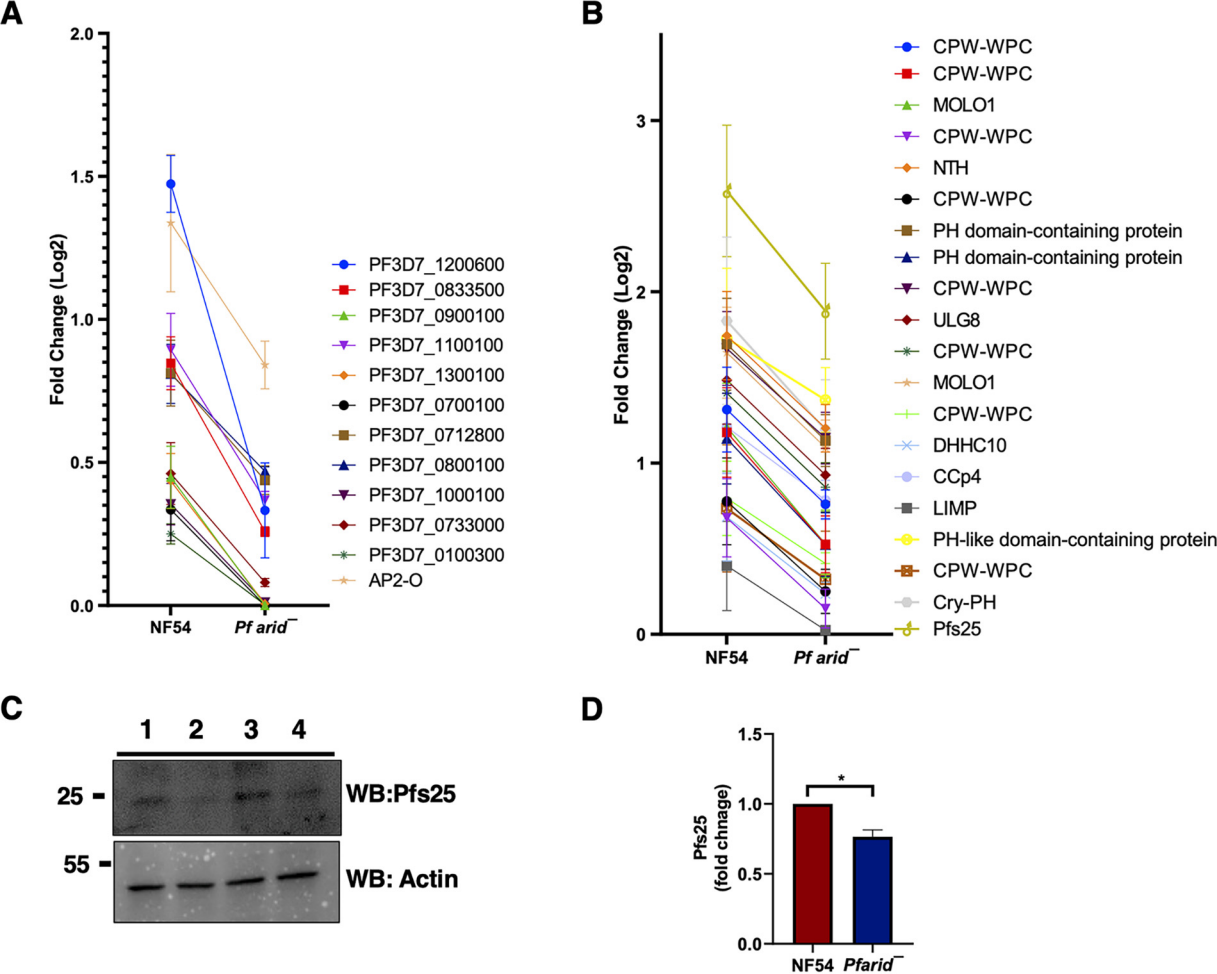
*Pf*ARID regulates expression of the PfAP2-O and PfHMGB2 target genes. (A) Graph showing DEGs related to AP2-O and its *VAR* targets. Log_2_ fold changes are indicated. (B) Graph showing DEGs related to PfHMGB2 regulated genes. Log_2_ fold changes are indicated. (C) Western blot analysis of Pfs25 expression in WT *Pf*NF54 and *Pfarid*^–^ gametocytes (upper panel). β-Actin was used as the loading control (lower panel). 1,3-WT *Pf*NF54; 2,4-*Pfarid*^–^ gametocytes.

Interestingly, three members of the ApiAP2 transcription factor family, including AP2-O, were also downregulated in *Pfarid*^–^ gametocytes, while AP2-L was upregulated (Data set S1A). PfAP2-O has been shown to bind upstream regions of *var* genes (48) and regulate their transcription (49). It also regulates sexual stage development and parasite transmission to the mosquito vector (49). A comparative analysis of DEGs from RNA-seq data with known PfAP2-O target genes (48, 49) revealed downregulation of numerous PfAP2-O target genes in *Pfarid*^–^ (Fig. 5A and Data set S1G), indicating *Pf*ARID-mediated regulation of PfAP2-O transcription factor function.

The downregulation of transcripts encoding P. falciparum orthologs of the P. berghei crystalloid proteins in *Pfarid*^–^ gametocytes was interesting. (Fig. S3D and Data set S1H). To identify a possible link between *Pf*ARID and crystalloid component-encoding genes, we searched published studies for chromatin regulators and other proteins which might regulate the expression of these genes. A high-mobility group box (HMGB) protein, HMGB2 has been demonstrated to control expression of several ookinete/oocyst-specific gene products in P. yoelii (50). Microarray analyses on *Pyhmgb2*^–^ parasites (asexual and gametocyte mix) revealed 30 genes to be downregulated, out of which 12 are expressed in ookinete/oocyst stages (50). Another study in P. berghei reported that these 12 ookinete/oocyst-specific proteins are present in a complex with LCCL lectin adhesive-like protein 3 (LAP3) along with crystalloid proteins (51). Interestingly, *Pf*HMGB2 was downregulated in *Pfarid*^–^ gametocytes (Data set S1C). While *Pf*HMGB2 shows 100% identity with P. yoelii HMGB2 (*Py*HMGB2), attempts at disrupting the gene have failed (52). To establish a possible link between *Pf*ARID and *Pf*HMGB2, we compared *Py*HMGB2 crystalloid gene targets (50) with those downregulated in *Pfarid*^–^. This analysis revealed common gene expression perturbations between these two data sets (Fig. 5B). *Pf*s25, which is an activated gametocyte/gamete and ookinete protein, was also among downregulated DEGs in *Pfarid*^–^ gametocytes (Data set S1C). Its ortholog *Py*s25 was also shown to be downregulated in *Pyhmgb2*^–^ gametocytes (50). We performed protein expression analysis for *Pf*s25 in WT *Pf*NF54 and *Pfarid*^–^ gametocytes via Western blotting, which revealed that *Pf*s25 levels were indeed reduced in *Pfarid*^–^ gametocytes (Fig. 5C and Fig. 5D).

## DISCUSSION

**FIG 6:**
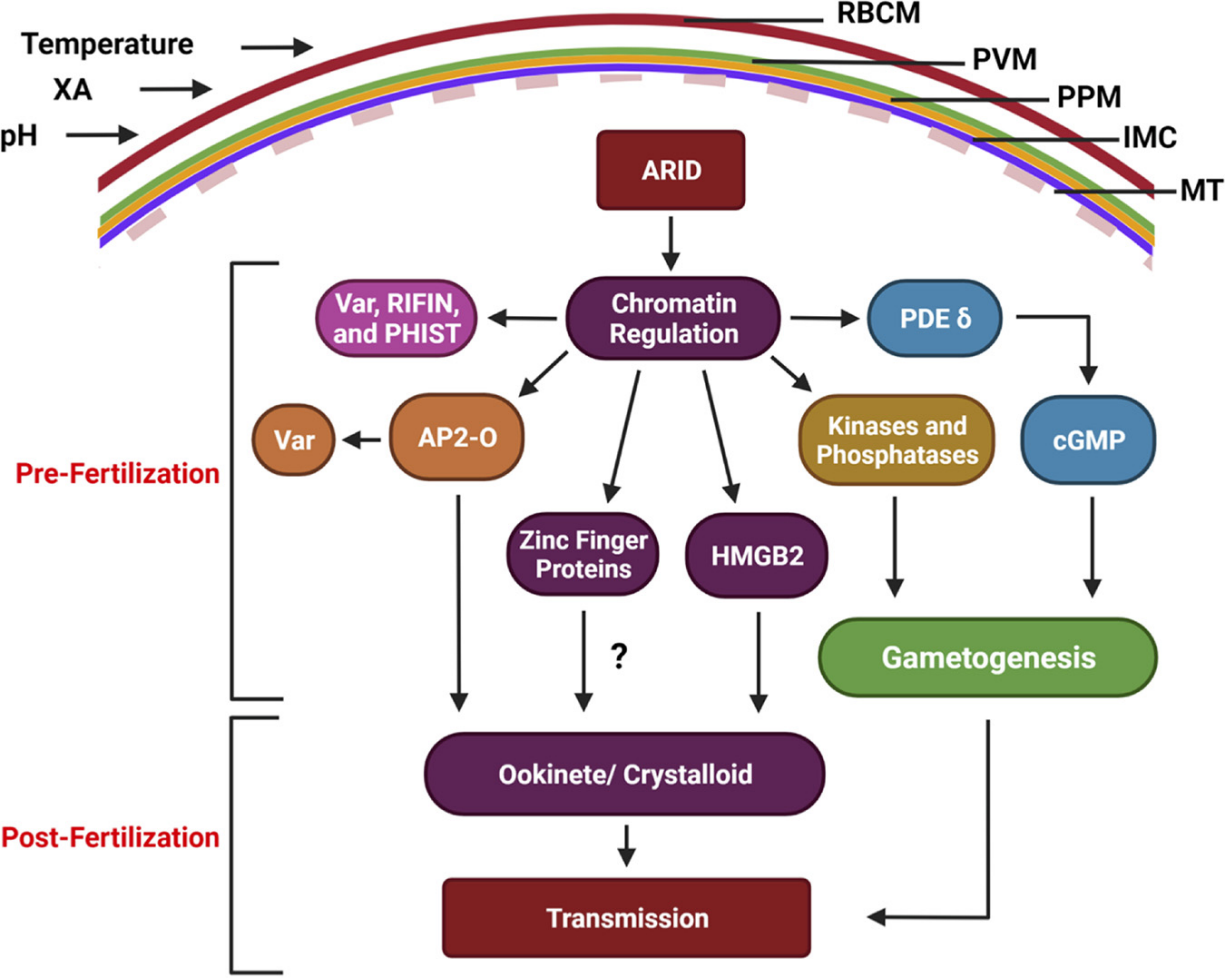
A model for *Pf*ARID function. *Pf*ARID regulates the stage V gametocyte chromatin landscape, likely as part of a chromatin remodeling complex. This in part controls expression of AP2-O, HMGB2, and zinc finger proteins (ZFPs), which in turn regulate heterochromatinized genes (*VAR*, *RIFIN*, *PHIST*), and ookinete/crystalloid-specific genes. ARID also controls expression of signaling molecules (kinases and phosphatase) which are known to regulate parasite functions. In addition, *Pf*ARID regulates expression of PDEδ, which in turn regulates cGMP levels and, thereby, gametogenesis. Collectively, these molecules regulate male gamete formation, female gamete fertility, and parasite infection of the mosquito vector.

Differentiation of a small subset of asexually replicating *Plasmodium* parasites into fertilization-competent gametes is a critical step for continuation of the parasite life cycle. The gametocytes taken up by the mosquito vector during a blood meal are rapidly activated to form gametes. For this, male gametocytes undergo three rounds of rapid DNA replication and each form eight flagellated male gametes (exflagellae). Female gametocytes undergo a marked reduction in cytoplasmic density and nuclear changes to each form a single female gamete. Gametocytogenesis and gametogenesis are critical steps and bottlenecks in the parasites’ life cycle. The well-studied ApiAP2 family protein AP2-G functions in the initiation of the transcriptional program that regulates the onset of gametocytogenesis (18, 53), but factors that regulate gene expression to drive gametogenesis and fertilization competence remain largely unknown. Our study demonstrates an essential regulatory role of the ARID domain-containing protein *Pf*ARID in microgametogenesis and macrogamete fertility, as well as postfertilization events that are transcriptionally established in mature gametocytes (Fig. 6).

The ARID family proteins ARID1A/ARID1B (also known as BAF250a/BAF250b) are part ATP-dependent chromatin remodeling complexes such as BAF (BRG1/BRM-associated factor or mammalian SWI/SNF) complexes in human cells (22). ARID proteins also form a complex with histone deacetylases in plants and regulate sperm cell formation (54). ARID complexes regulate maintenance of open chromatin at enhancer elements to drive gene expression programs in a developmentally regulated and cell context-specific manner (22). *Plasmodium* contains components of chromatin-modifying proteins (55), and genome-wide nucleosome mapping has indicated that chromatin remodeling might be an important mechanism of gene regulation in these parasites (56). However, no chromatin remodeling complexes or BAF complex components have been characterized in P. falciparum. Using CRIPSR/Cas9-based gene editing, we generated *Pfarid*^–^ parasites and demonstrated that *Pf*ARID is critical for microgametogenesis but also regulates fertility of macrogametes. Although *Pf*ARID is expressed in the asexual erythrocytic stages, the deletion of the gene did not cause an overt phenotypic defect in these parasite stages. We thus analyzed the perturbation of the transcriptome in the sexual stages and found that *Pf*ARID is regulating expression of genes that drive gametogenesis, of gene products that form signaling cascades, and of gene products critical for the ookinete crystalloid organelle and expression of multigene families.

*Pf*ARID possesses multiple NLS signals, a LXCXE motif and two TM domains. In agreement with the NLS prediction, we showed that *Pf*ARID displayed a nuclear localization in both asexual and sexual stages. An Internal bipartite NLS has been shown to regulate mammalian ARID1A nuclear localization (57). The presence of TM domains in *Pf*ARID is unique and intriguing. Nuclear envelope transmembrane proteins (NETs) have been described to control the cell cycle (58) and organize spatial control of the genome (59), but no ARID family protein is known to possess TM domains (28). We have also shown that *Pf*ARID localized in close proximity to nuclear pore complexes, further suggesting an association with the nuclear envelope. It is possible that its two predicted TM domains anchor *Pf*ARID to the nuclear envelope. *Pf*ARID also contained an LXCXE motif, which in other proteins has a role in facilitating interaction with the retinoblastoma (RB) tumor suppressor (29). ARID4A is an RB-binding protein and regulates cell cycle progression in a variety of organisms (60–62). ARID4A and ARID4B are involved in the control of male fertility by acting in the RB pathway (63). An RB pathway and its components have, however, not been identified in *Plasmodium.* It is thus possible that other parasite proteins might bind to *Pf*ARID via the LXCXE motif and regulate its function. The ARID domain of *Pf*ARID displayed high structural similarity to the ARID domain of human ARID3a, which implicates it in transcriptional regulation, as ARID3a has been shown to play a role in regulation of transcription factors associated with hematopoietic lineage decisions and regulation of myeloid and B lineage pathways (30).

Our gene deletion analysis showed that *Pf*ARID is not required for asexual blood-stage replication or gametocyte development but uncovered its critical role in male gamete formation, specifically the formation of flagellated microgametes. Male *Pfarid*^–^ gametocytes also did not show the typical morphological changes that lead to the formation of a spheroid infected RBCs upon activation. In contrast, we observed no discernible defect in *Pfarid*^–^ macrogamete formation. The *Pfarid*^–^ genetic crosses we performed with transgenic lines producing either fertile microgametes (6) or macrogametes (35) confirmed a completely penetrant male defect but, surprisingly, also showed that *Pf*ARID is required for fertility of female gametes. A recent study describing screening for fertility-related genes in the rodent malaria parasite P. berghei, showed that the P. berghei ARID (*Pb*ARID) ortholog named MD4 (PBANKA_0102400) is involved in fertility of male gametes only (42). Thus, ARID might differ in its sex-specific functions among different malaria parasite species.

Given the nuclear localization of *Pf*ARID, its colocalization with the euchromatic acetylated histone marker H3K9Ac and the severe gamete defects of *Pfarid*^–^ parasites, we hypothesized that *Pf*ARID might be controlling gametogenesis and fertility by regulating stage V gametocyte gene expression. Indeed, comparative transcriptome analysis of *Pf*NF54 WT and *Pfarid*^–^ parasites using RNA-seq identified 411 differentially expressed genes (DEGs) which were downregulated in parasites lacking *Pf*ARID as well as 78 upregulated DEGs. Likely, these DEGs might be directly regulated by *Pf*ARID but also indirectly by the perturbation of transcription factor expression. We observed downregulation of key gametogenesis-regulating gene products, including *PfPDEδ*. Previous work has demonstrated that *Pf*PDEδ activity, optimal cGMP levels, and cGMP-dependent kinase PKG are required for microgametogenesis and liberation of the male gametes from the infected RBC during exflagellation (36, 64). We observed an increase of cGMP levels in *Pfarid*^–^ parasites, which would severely impact microgametogenesis. Also, DEGs observed in *Pfarid*^–^ parasites may represent targets of cGMP-*Pf*PDEδ-mediated homeostasis and signaling and other parasite proteins which may have a role in microgametogenesis. Indeed, expression of GTP cyclohydrolase 1 (GCH1) was elevated in *Pfarid*^–^ gametocytes (Data set S1B). GCH1 catalyzes conversion of GTP into DHNTP (7,8-dihydroneopterin triphosphate) and is critical for parasite transmission in P. berghei (43). Other DEGs that were downregulated in the absence of *Pf*ARID, such as NIMA-related kinase 3 (NEK3), cell division control protein 6 (CDC6), DLC7, centrin 1, and centrin 2, as well as CDKr show very high expression in late-stage gametocytes (PlasmoDB). These proteins are currently uncharacterized in P. falciparum, but P. berghei orthologs of some of these proteins have been shown to play a role in sexual development (65). It would thus be important to study their function during P. falciparum microgametogenesis. Interestingly, transcripts encoding several signaling molecules, such as kinases and phosphatases, were also downregulated in *Pfarid*^–^, indicating a potential perturbation of phosphorylation of parasite proteins in stage V gametocytes. Kinases and phosphatases have been shown to play a role in gametogenesis and transmission to the mosquito vector in both P. falciparum (6, 64, 66) and P. berghei (67, 68).

Surprisingly, another group of DEGs downregulated in *Pfarid*^–^ parasites encode ookinete/crystalloid-related genes. Crystalloids are unique organelles of the ookinete stages, which develop after fertilization from the zygote and are built to invade the mosquito midgut to form oocysts. They appear as clusters of tightly packed electron-dense spherical units in the ookinete cytoplasm (69) and are involved in sporogony, the formation of sporozoites within the oocyst (70). Recent studies in P. berghei have identified components of crystalloids, including LCCL lectin adhesive proteins (LAPs), CPW-WPC family proteins, secreted ookinete proteins (SOPs), a palmitoyl-S-acyl transferase (PAT) protein named DHHC10, NAD(P) transhydrogenase (NTH), a multipass transmembrane protein that generates NADPH, and several PH domain-containing proteins (51). We found that a number of transcripts, including those for DHHC10, NTH, CPW-WPC family proteins, and LAP6 were downregulated in *Pfarid*^–^ parasites. This might be indirectly driven by the observed downregulation of *Pf*HMGB2 in *Pfarid*^–^ parasites, a member of the high mobility group box (HMGB) family, which in other organisms actively participates in chromatin remodeling by increasing nucleosome sliding and accessibility of the chromatin (71). Previous studies have implicated *Py*HMGB2 in regulating expression of the orthologous genes in P. yoelii (50). Interestingly, many of the HMGB2-regulated gene transcripts are then translationally repressed by the development of zygote inhibited (DOZI) mRNA storage complex (72). This complex represses premature translation of mRNAs in gametocytes that encode proteins which function postfertilization. Thus, *Pf*ARID in part regulates the expression of mRNAs that are stored and translationally repressed in gametocytes and are translated only after fertilization to drive infection of the mosquito vector.

Transcriptional regulation in *Plasmodium* is thought to mainly involve members of the ApiAP2 transcription factor family. We found that some ApiAP2 family members such as AP2-O were downregulated in *Pfarid*^–^ parasites. AP2-O has been previously implicated in regulating expression of genes involved in parasite transmission to the mosquito vector (49). This suggests an additional level of complexity to *Pf*ARID function, as it might not only directly regulate accessibility of transcription factors to DNA but might also directly regulate expression levels of transcription factors.

Lastly, gametocytes lacking *PfARID* also showed dysregulation (both upregulated and downregulated) of a large number of heterochromatin-associated multigene families such as *VAR* (73), *RIFIN* (74), and *PHISTa/b/c* (75), which are known to regulate cytoadherence of infected RBCs, immune escape, and other parasite/host interactions, mainly in the parasites’ asexual blood stages. These gene families are, however, also expressed in gametocytes (76–79). *VAR* gene expression encoding PfEMP1 proteins might continue to provide variant antigen expression and immune escape in gametocytes during maturation (76), and PHIST family proteins are exported during gametocytogenesis (77) and control infected RBC rigidity (80). Host cell deformability and rigidity change late in gametocytogenesis and are possibly critical factors in gametocyte transmission to the mosquito vector (81). Since we have shown that *Pf*ARID is also expressed in asexual blood stages, but its deletion did not result in a parasite growth defect, it will be of interest to analyze the perturbation of multigene family expression in these stages in the future.

Our demonstration that *Pf*ARID serves an essential role in driving microgametogenesis and macrogamete fertility via regulation of gene expression constitutes a critical entry point for understanding the regulation of P. falciparum gamete formation and fertilization competence on the molecular level. Since ARID proteins in other organisms are part of BAF chromatin-remodeling complexes (22), the identification *Pf*ARID will enable the isolation of equivalent complexes in *Plasmodium*. A fuller understanding of *Pf*ARID-mediated chromatin regulation might also inform novel transmission-blocking interventions against malaria parasites.

## MATERIALS AND METHODS

### Reagents and primary antibodies.

All molecular biology reagents and oligonucleotides were purchased from MilliporeSigma, USA, unless otherwise stated. All oligonucleotides were purchased from Integrated DNA Technologies, Inc. (IDT), USA. The following primary antibodies and antisera and dilutions were used: mouse α-P230p (1:100, kindly gifted by Kim Williamson, Uniformed Services University of the Health Sciences, USA) α-*Pf*g377 (1:500, kindly gifted by Pietro Alano at Istituto Superiore di Sanità, Italy), mouse α-tubulin antibody (1:200, Sigma-Aldrich, catalog [cat.] no. T5168), rat α-mCherry antibody (1:200, Thermo Scientific cat. no. M11217, clone 16D7), α-NUP116 (1:100, rabbit, kindly gifted by Artur Scherf at Institut Pasteur, France). The following reagents were obtained through BEI Resources, NIAID, NIH: hybridoma 4B7 anti-*Pf*s25-kilodalton gamete surface protein (*Pf*s25), MRA-315, contributed by Louis H. Miller and Allan Saul and α-*Pf*s25 (1:1, mouse).

### P. falciparum culture and transfection.

P. falciparum parasites (WT *Pf*NF54 and *Pfarid*^–^) were cultured as asexual blood stages according to standard procedures and received complete RPMI medium supplemented either with 0.5% AlbuMAX II (Thermo Scientific) medium or 10% (vol/vol) human serum every 24 h. *In vitro* gametocytes were generated using O^+^ human RBCs (Valley Biomedical, Virginia, USA) and O^+^ human serum (Interstate Blood Bank, Tennessee, USA) using methods published elsewhere (32).

Oligonucleotide primers used for the creation and analysis of P. falciparum *Pfarid*^–^ and *Pf*ARID^mCherry^ parasites are detailed in Table S1. Deletion of *PfARID* (PlasmoDB identifier gene PF3D7_0603600) was achieved based on the previously reported CRISPR/Cas9 strategy. Gene deletion was confirmed by a set of genotyping PCRs (Fig. S2B). Two individual clones for *Pfarid*^–^ (clones 4E and 6A) were used for phenotypic characterization.

10.1128/mbio.00578-22.3 TABLE S1 Oligonucleotides used in the study. Download Table S1, DOCX file, 0.02 MB. Copyright © 2022 Kumar et al. 2022 Kumar et al. https://creativecommons.org/licenses/by/4.0/ This content is distributed under the terms of the Creative Commons Attribution 4.0 International license.

### Measurement of asexual blood-stage growth and gametocyte development.

To compare asexual blood stage replication and growth between the WT *Pf*NF54 and *Pfarid*^–^ parasites, synchronized parasites were set up at an initial ring stage parasitemia of 1% and cultured in 6-well plates. Thin smears were prepared at 48 and 96 h. For preparation of Giemsa-staining, parasitemia was scored per 1,000 erythrocytes.

To compare gametocyte formation between WT *Pf*NF54 and *Pfarid*^–^, gametocytes were cultured as described above. Parasites were removed on day 15 of *in vitro* culture for preparation of Giemsa-stained thin blood smears, and gametocytemia was scored per 1,000 erythrocytes.

### Indirect immunofluorescence.

For IFAs on gametocytes and exflagellating gametes, thin smears were prepared on Teflon-coated slides and fixed with 4% paraformaldehyde/0.0025% glutaraldehyde solution for 30 min. Slides were kept in a humidity chamber for each step. Fixed parasites were washed twice with phosphate-buffered saline (PBS) and permeabilized using 0.1% Triton X-100/PBS solution for 10 min. Parasites were washed twice with PBS for 5 min each and blocked with 3% bovine serum albumin (BSA)/PBS for 45 min. Primary antisera in 3% BSA/PBS were added to the parasites, and the slides were incubated at 4°C. Antigens were visualized using anti-species antibodies. Images were obtained using a ×100 1.4-numerical aperture (NA) objective 90 (Olympus) on a Delta Vision Elite high-resolution microscope (GE Healthcare Life Sciences).

### Comparative RNA-Seq and data analysis.

RNA-seq methodology was adapted from previous articles with modifications (82, 83). On day 15 of gametocyte development, stage V gametocytes were harvested using saponin lysis. The RNA preparation, library preparation, and RNA-seq analysis were done at Azenta/Genewiz, USA. Total RNA from saponin-lysed parasites was extracted using TRIzol (Invitrogen) and a Qiagen RNA-extraction kit. Following RNA isolation, total RNA integrity was checked using a 2100 Bioanalyzer (Agilent Technologies, Santa Clara, CA, USA). RNA concentrations were measured using the NanoDrop system. rRNA was removed from total RNA using an Illumina Ribo Zero Gold for human/mouse/rat kit. The libraries were multiplexed and clustered on one lane of a flow cell and loaded on an Illumina HiSeq platform according to the manufacturer’s instructions. After the quality of the raw data was investigated, sequence reads were trimmed to remove possible adapter sequences and nucleotides with poor quality using Trimmomatic v.0.36. The trimmed reads were mapped to the Plasmodium falciparum reference genome using the STAR aligner v.2.5.2b. BAM files were generated because of this step. Unique gene hit counts were calculated by using the Counts feature from the Subread package v.1.5.2. R software v.3.4.1 was used when executing DESeq2 analysis for DEG identification and graphic tools. All the analyses were performed with default parameters; DEGs were defined as genes with an absolute log_2_ fold change (log2FC) of >1 and adjusted *P* value of <0.05.

Gene Ontology term enrichment analyses were carried out with Cytoscape v.3.9.0 (84) with the BiNGO plugin (85). Gene Ontology (GO) terms for P. falciparum genes were downloaded from the PlasmoDB database. GO terms from all three categories were fetched from this and used as input against all the known GO terms in the BiNGO plugin. The hypergeometric distribution test was performed at a *P* value of ≤0.05 with Bonferroni correction. The network of enriched GO terms thus obtained was reported as the result.

### Measurement of cGMP levels.

The assay for determining cGMP levels in gametocytes was performed using the cGMP enzyme immunoassay (EIA) kit (cat. no. 581021; Cayman Chemical) following the manufacturer’s instructions. Gametocytes for the assay were purified on a Percoll gradient to get rid of uninfected RBCs, and gametocyte extracts were prepared by two rounds of freezing on dry ice-ethanol, thawing on ice, and passaging through a 21-gauge needle from the same number of gametocytes for each line. Equal volumes of extract from WT *Pf*NF54 and *Pfarid*^–^ gametocytes were used to assay for cGMP.

### Statistical analysis.

All data are expressed as the mean ± standard deviation (SD). Statistical differences were determined using one-way analysis of variance (ANOVA) with the *post hoc* Bonferroni multiple-comparison test or unpaired two-tailed Student’s *t* test, as indicated. Values of *P* < 0.05 were considered statistically significant. Significances were calculated using GraphPad Prism 8 and are represented in the figures as follows: ns, not significant, *P* > 0.05; *, *P* < 0.05; **, *P* < 0.01; ***, *P* < 0.001.

### Data and material availability.

All the correspondence and request for materials used in these studies should be addressed to the corresponding authors.
