# Supplementary figures and images for: PfARID Regulates P. falciparum Malaria Parasite Male Gametogenesis and Female Fertility and Is Critical for Parasite Transmission to the Mosquito Vector
Source: mBio. 2022 May 31;13(3):e00578-22. doi: 10.1128/mbio.00578-22 (PMC9239086; doi:10.1128/mbio.00578-22)

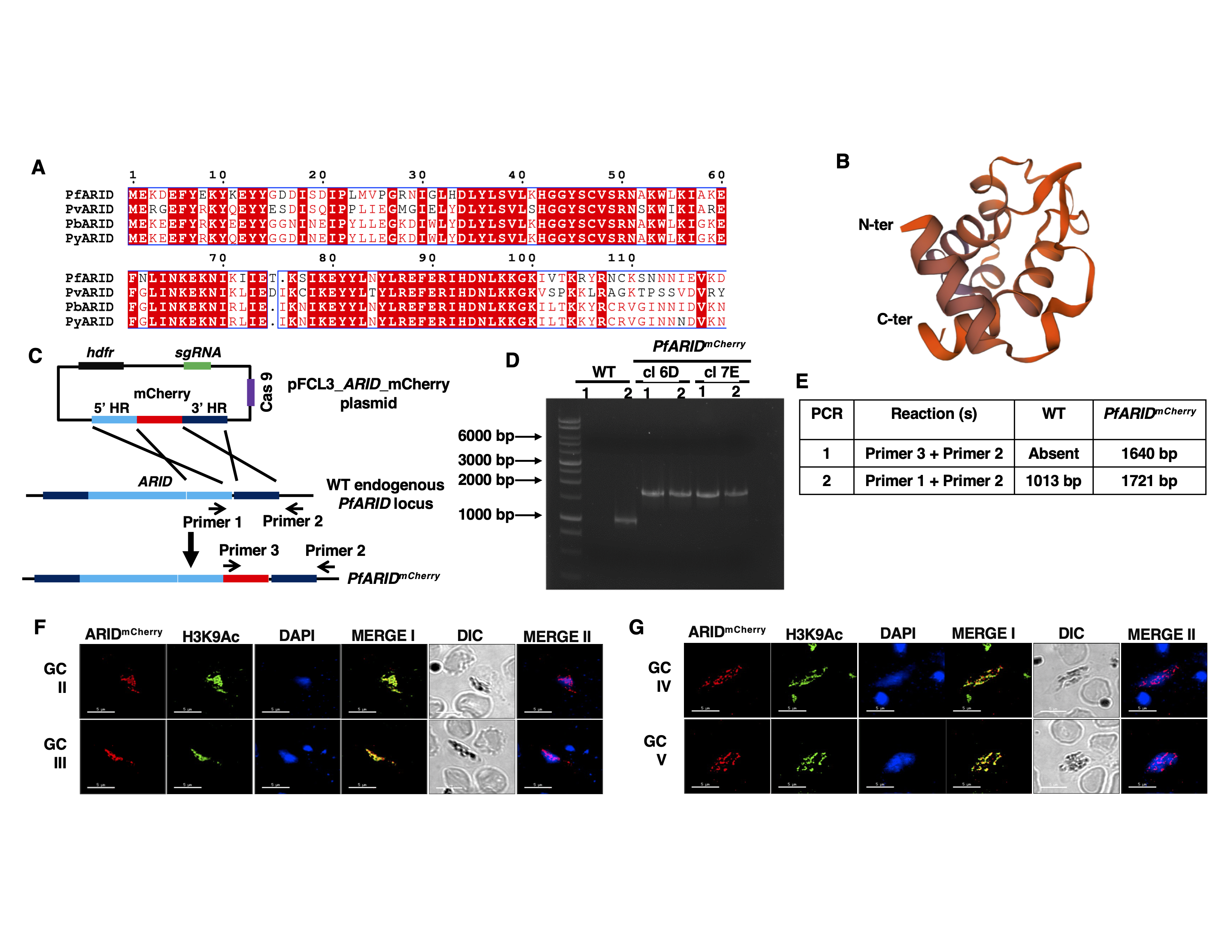

Supplement: FIG S1 [file mbio.00578-22-s0002.tif]

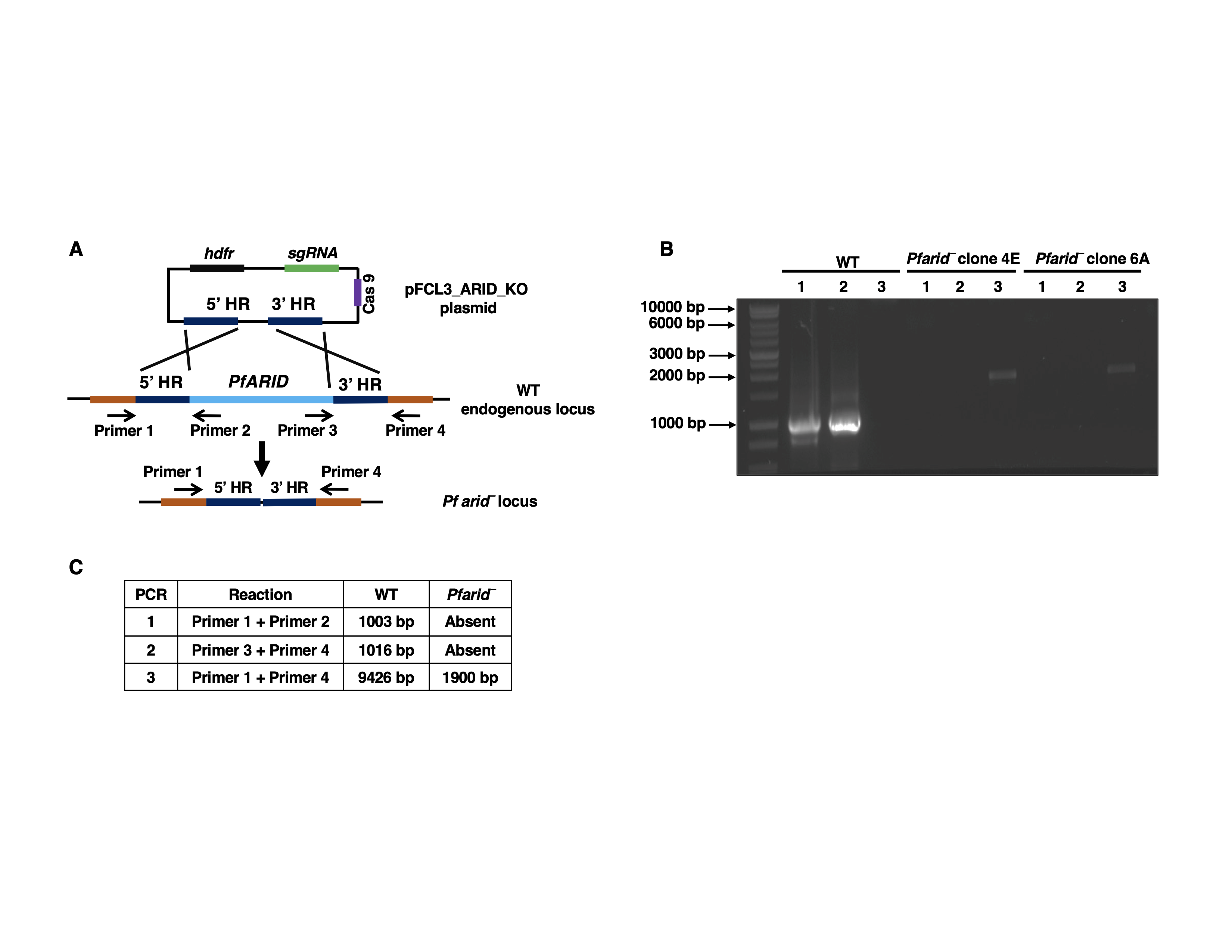

Supplement: FIG S2 [file mbio.00578-22-s0003.tif]

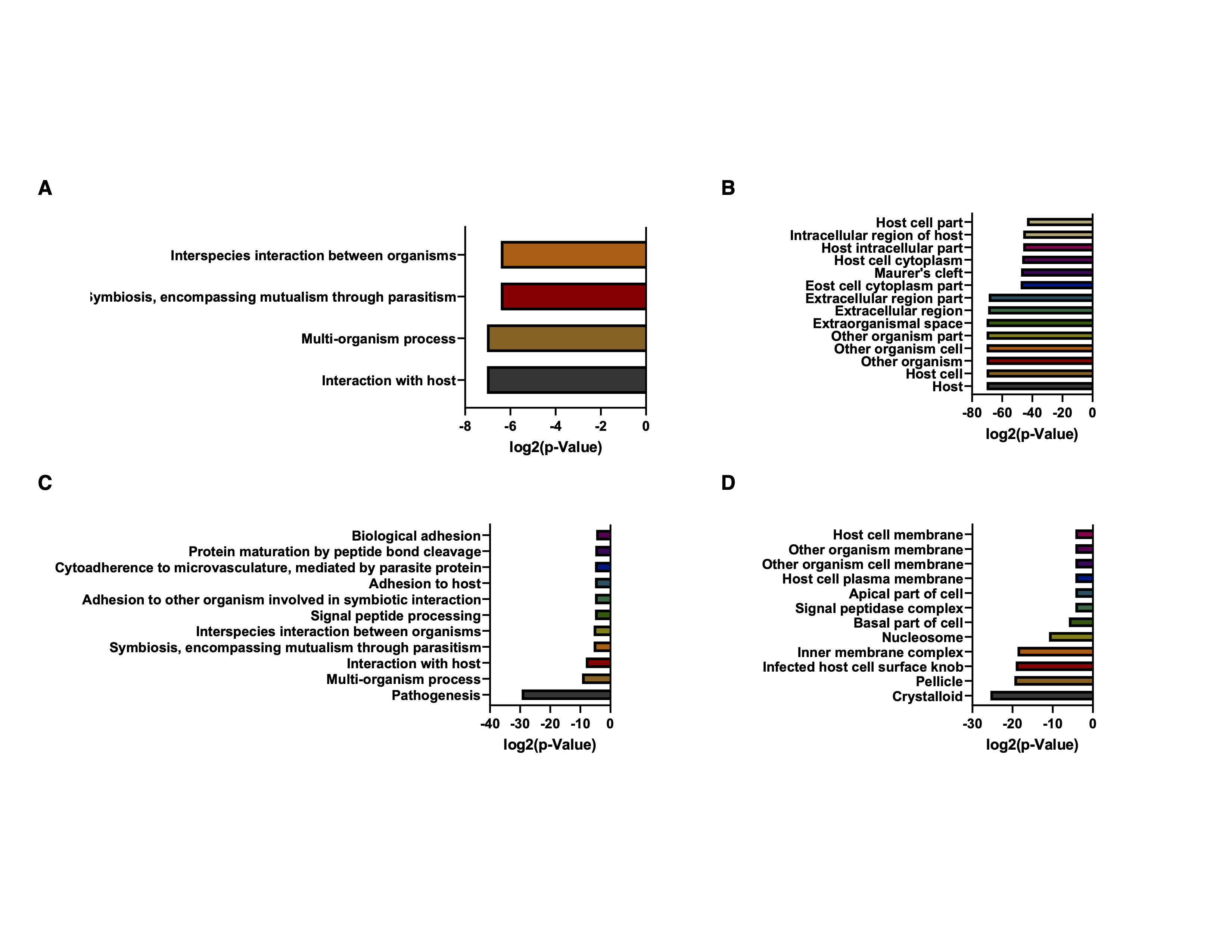

Supplement: FIG S3 [file mbio.00578-22-s0004.tif]

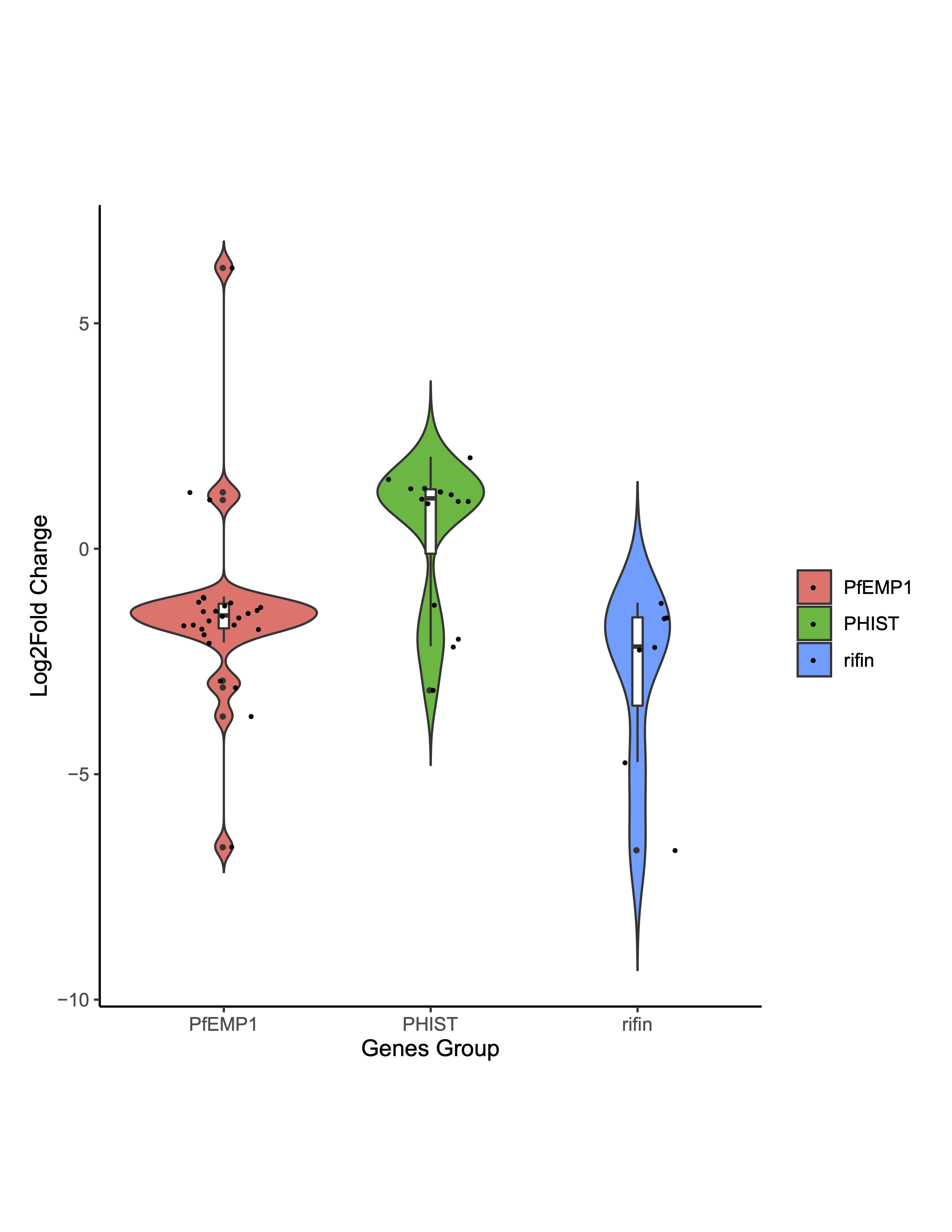

Supplement: FIG S4 [file mbio.00578-22-s0005.tif]
