# Supplementary material for: PfARID Regulates P. falciparum Malaria Parasite Male Gametogenesis and Female Fertility and Is Critical for Parasite Transmission to the Mosquito Vector
Source: mBio. 2022 May 31;13(3):e00578-22. doi: 10.1128/mbio.00578-22 (PMC9239086; doi:10.1128/mbio.00578-22)
Supplement: TABLE S1 [file mbio.00578-22-s0006.docx]

**Table S1: Oligonucleotides used in the study:**

| **Oligonucleotides used for generation of *Pfarid¯* parasites** | |
| --- | --- |
| **Oligo** | **Forward (5’-3’)** |
| PfARID 5’Homo For | T**GCGGCCGC**TCATAAAATAAGCTCAAATAGTTAAAAAGGC |
| PfARID 5’Homo Rev | CCAACCCGGGTATAGGCGCGCCTCCCTTACAGAAAAAAAAAAATTCCTAACC |
| PfARID 3’Homo For | AGGCGCGCCTATACCCGGGTTGGATATTCTCCCCAATAAATAAATAAATAAATATATAC |
| PfARID 3’Homo Rev | TAA**GTCGAC**GTTGGTGTTGTTTTCTGGTTTGATACAT |
| PfARID Guide 1 For | **TATT**GTTTCGACCAGGAACCATTAA |
| PfARID Guide 1 Rev | **AAAC**TTAATGGTTCCTGGTCGAAAC |
| PfARID Guide 2 For | **TATT**GTATATTACCTGTGTGATTAG |
| PfARID Guide 2 Rev | **AAAC**CTAATCACACAGGTAATATAC |
| PfARID Geno5 For | GATACTATTTGTCTTTTTCTATAAATTATATAAATCC |
| PfARID Geno5 Rev | TTTCGACCAGGAACCATTAAAGG |
| PfARID Geno3 For | TCAAAGTTGGTAAAGGTTGAAAAGATTA |
| PfARID Geno3 Rev | GGAGAAAGCAATTGGTTAGAAGGAG |
| **Oligonucleotides used for generation of *PfARID^mCherry^* parasites** | |
| PfARID 5GBlock GFP For | ATACTAGTATAGCTAGCTATCAAAAAAATGACGACAACCAATT |
| PfARID 5GBlock GFP Rev | TCTTCTTCTCCTTTTGAAACTTTAATCTTTTCAACCTTTACC |
| PfARID 3G Block GFP_For | GATGAATTATATAAATAAGCGGCCGCCACTTATAAAGTGAATTCTGTATAG |
| PfARID 3G Block GFP Rev | CGGTCATGAATTCCTCGAGCGGCCGCGTTGGTGTTGTTTTCTGG |
| PfARID Guide5 For | **TATT**GAGAATATAAAAAAATGAAA |
| PfARID Guide5 Rev | **AAAC**TTTCATTTTTTTATATTCTC |
| mCherry For | ATC**GTCGAC**GTTTCAAAAGGAGAAGAAGATAATATGG |
| PfARID Geno3 For | TCAAAGTTGGTAAAGGTTGAAAAGATTA |
| PfARID Geno3 Rev | GGAGAAAGCAATTGGTTAGAAGGAG |
